# Supplementary material for: Plasma FIB milling for the determination of structures in situ
Source: Nat Commun. 2023 Feb 6;14:629. doi: 10.1038/s41467-023-36372-9 (PMC9902539; doi:10.1038/s41467-023-36372-9)
Supplement: Supplementary file 1 — Supplementary Information [file 41467_2023_36372_MOESM1_ESM.pdf]

Supplementary Figures

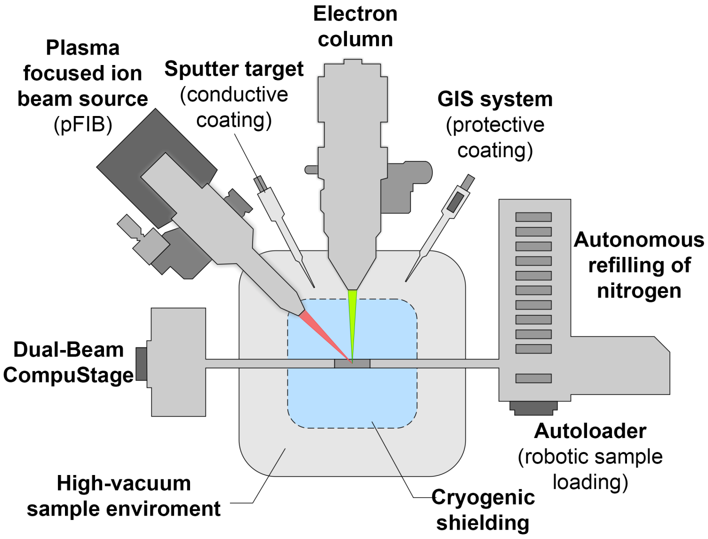

Supplementary Figure 1 - Schematic overview of the plasma FIB/SEM microscope used in this study. The microscope has several elements that make it amenable to lamella production on long time scales in an automated fashion. This includes the high-vacuum chamber (10^{-7} mbar), multi-specimen loading and automatic refilling with liquid nitrogen. The instrument is equipped with a Hydra-type focused ion beam column, which utilises xenon, argon, or oxygen gas sources for plasma generation. The CompuStage enables robust position of the sample within the co-incidence point with ~500 nm accuracy. Gas injection system (GIS) and sputter target enable protective layer and charge mitigation functionality to enable scanning electron imaging using the NiCol SEM column.

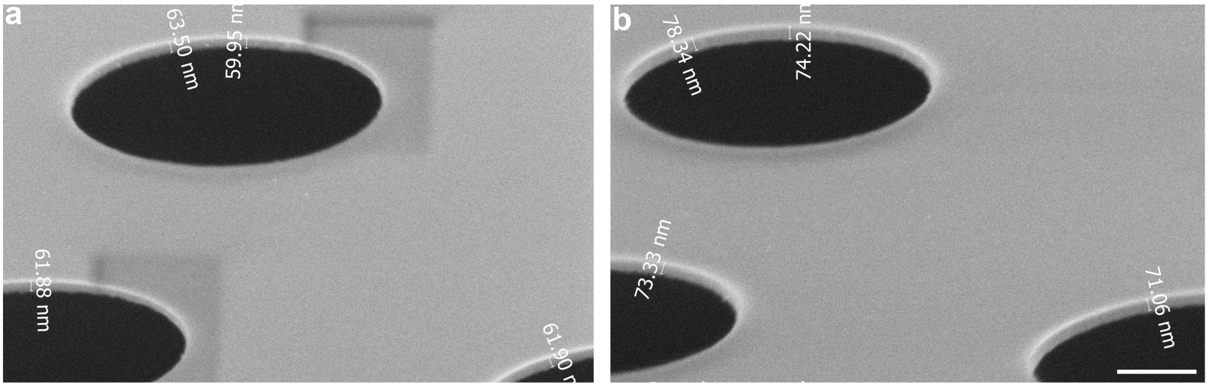

Supplementary Figure 2 - Ice growth over time in in the column of the plasma FIB/SEM microscope. SEM images of the same location of a TEM grid were acquired at 0 h (a) and 8 h (b) and the EM grid foil thickness measured before and after, corrected for the stage tilt. Four measurements were taken from one experimental repeat. This gave an average ice growth rate in the column of 1.6 nm/h. Scale bar: 500 nm. The pixel size for these images was 1.3 nm. Source data are provided as a Source Data file.

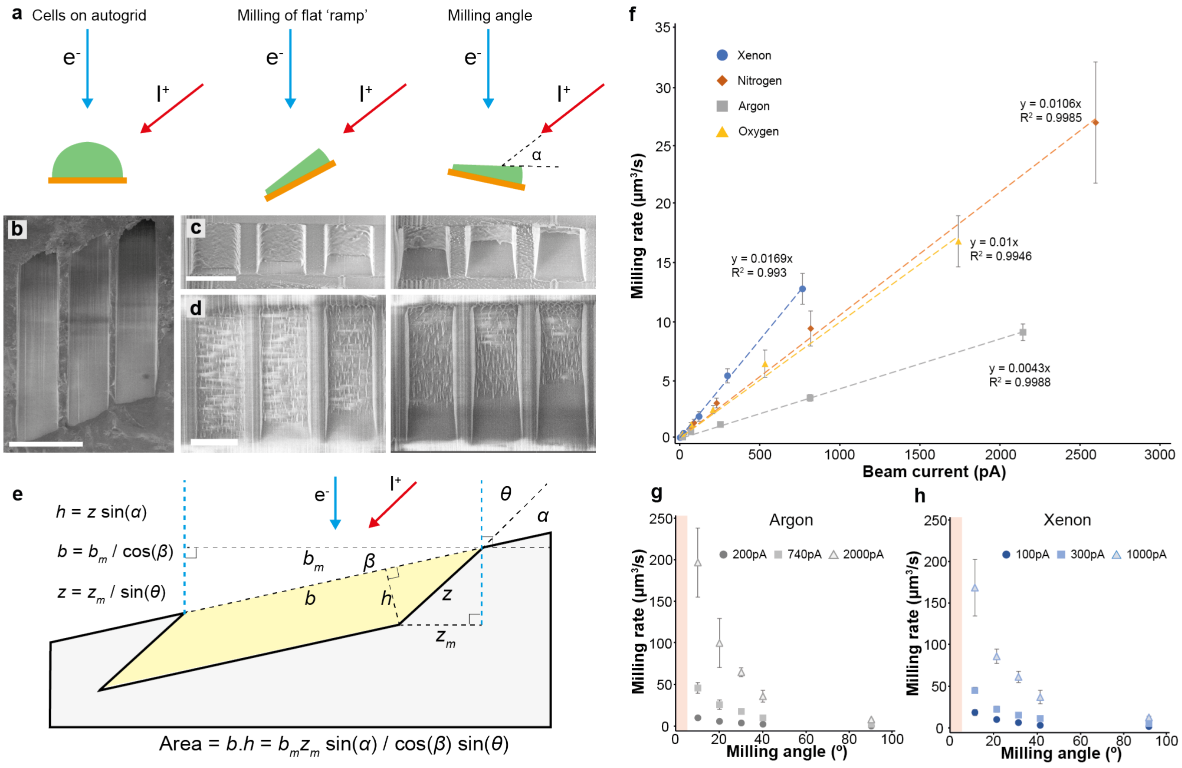

Supplementary Figure 3 – Determination of PFIB milling rates at 30 kV on vitrified yeast samples. (a) Cartoon showing milling rate measurement process, which involves milling a flat ramp **(b)** before tilting to present this fresh surface to the PFIB at a milling angle of alpha. Scale bar: 40 µm. Six rectangles of varying exposure time were then milled and repeated three times, giving 18 measurements for each condition. Representative images of milling angles of 90° and 20° are shown in **(c)** and **(d)** respectively. Scale bars: 5 µm. The depth of the milled trench increases in **(c)** and **(d)** from left to right, as a function of milling time. The volume was then calculated from measurement of the milled depth using the SEM images, as shown in **(e)** from which the milling rate could be calculated (see materials and methods). **(f)** Milling rate vs current for xenon, nitrogen, argon, and oxygen at a range of currents with a milling angle of 90°. The mean milling rate from n = 18 is plotted. The error bars are the standard deviation (Table 1). Source data are provided as a Source Data file. **(g)** and **(h)** show the milling rate as a function of milling angle for three different beam currents of argon and xenon respectively. The red area shows where the trend is expected to reduce back to zero but was not included as accurate measurement of such low angle mills was experimentally challenging. The mean milling rate from n = 18 is plotted. The error bars are the standard deviation (Table 2). Source data are provided as a Source Data file.

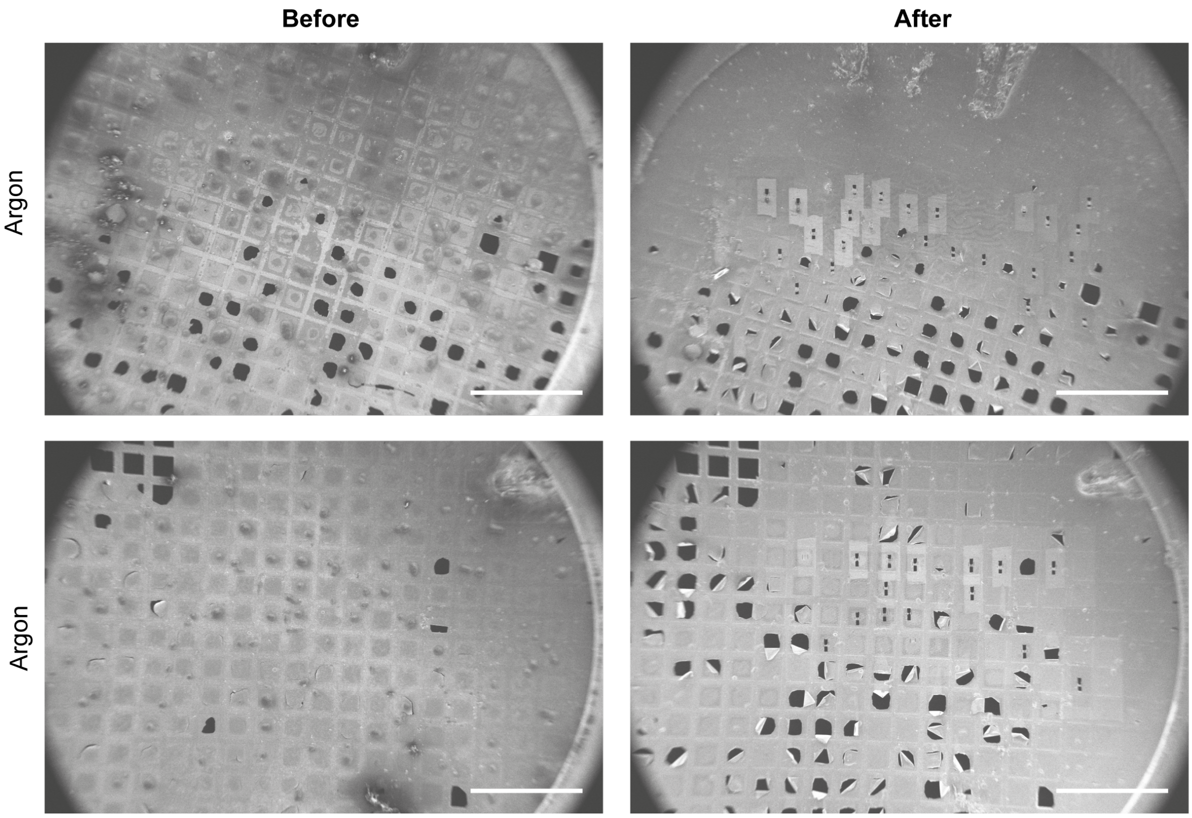

Supplementary Figure 4. SEM grid overviews before and after lamella fabrication. From three independent plasma FIB milling sessions using argon. Scalebar: 500 µm.

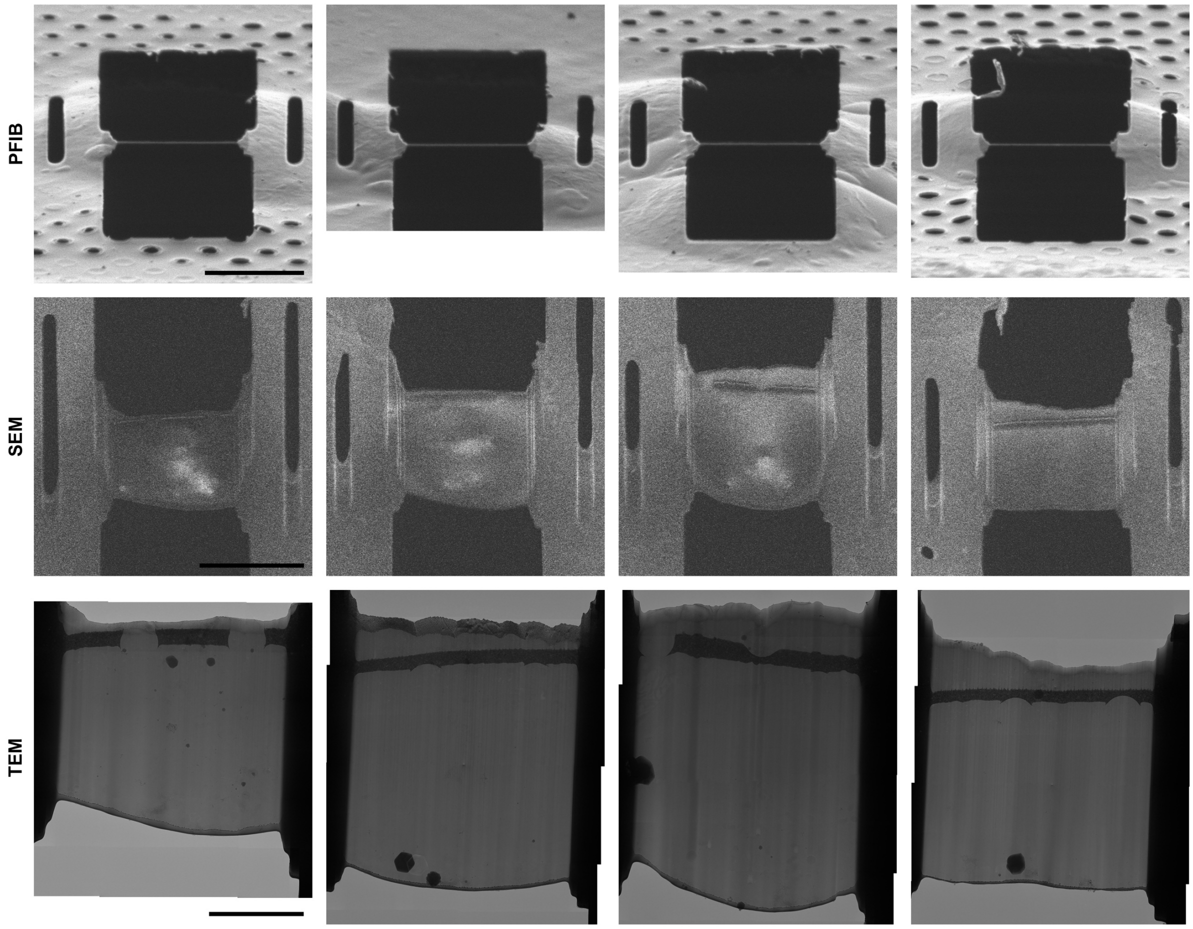

Supplementary Figure 5. SEM, PFIB and TEM overviews of lamellae. Lamellae created with automated milling using argon in three independent sessions. PFIB and SEM images were acquired directly after fine polishing and the matching low-dose tilescan overviews were acquired before tomography. Scalebars PFIB and SEM: 10 µm, scalebar TEM: 5 µm. Cells were grown on UltraAuFoil grids, with the gold foil visible as a dark horizontal stripe in the lamellae.

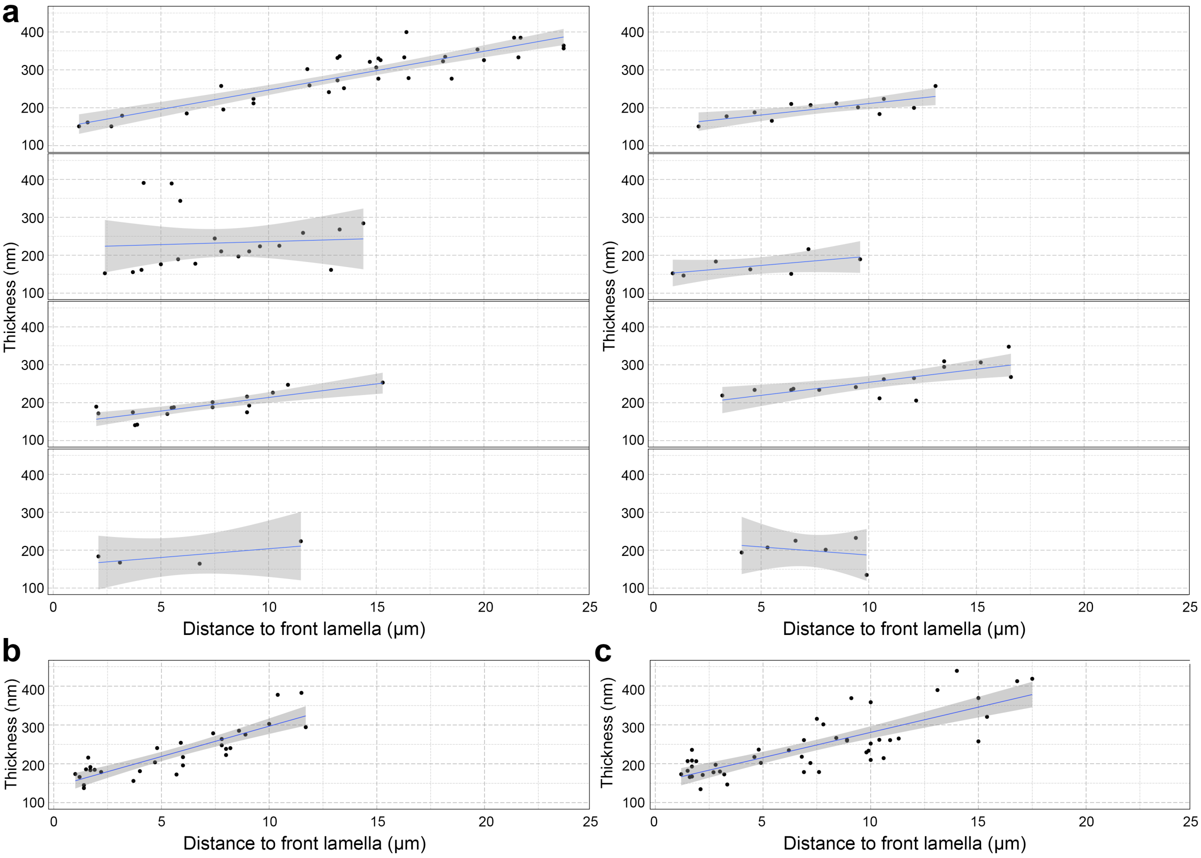

Supplementary Figure 6 – Distances from the edge of the front of the lamella for tomograms vs the measured thickness for a given tomogram. (a) Scatter plots for tomogram thickness and the distance to the front of the lamellae. A linear trend line (blue) is shown, with the 0.95 confidence interval for the trend line shown in grey. Plots are shown for 8 lamellae of the distribution of thickness against the distance form the leading edge of the lamella. The plots show the thickness profile of the lamella and indicate their flatness. (b, c) plots for all the tomograms acquired on the PFIB lamellae for two of the argon datasets were acquired on 11 and 16 different lamellae respectively. A linear trend line (blue) is shown, with the 0.95 confidence interval for the trend line shown in grey. Source data are provided as a Source Data file.

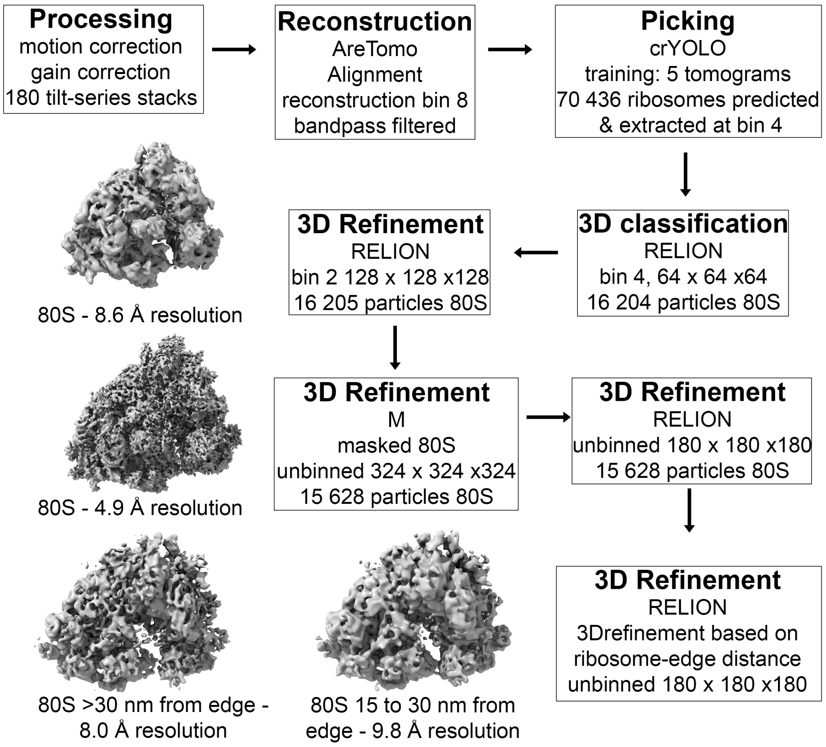

Supplementary Figure 7. Schematic overview of sub volume averaging workflow. All 180 tilt-series were processed, reconstructed and putative ribosomes were identified with crYOLO. Further 3D classification in RELION and subsequent 3D refinement in RELION and M yielded a 4.9 Å reconstruction of the 80S ribosome, and two separate reconstructions with a subset of the particles based on their distance from the lamella milling edge.

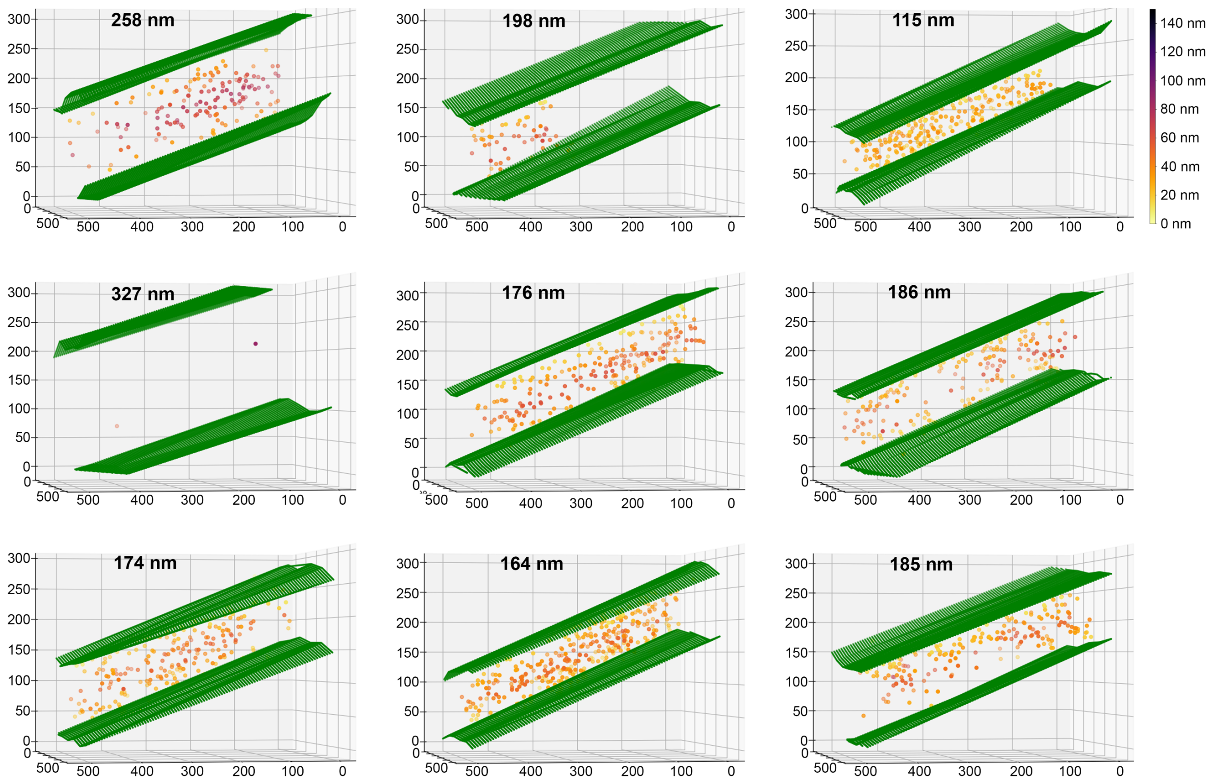

Supplementary Figure 8 – Distribution of ribosome locations within 9 representative tomograms. Graphical representation of the annotated lamella edges (green) of 9 different tomograms. Ribosomes used for sub-volume averaging (see Fig. 3) are shown, where the colour of the ribosome points are indicative of their distance from the edge (in nanometres). XYZ coordinates are indicated for 8 x down sampled tomograms (pixel size: 1.48 nm). Source data are provided as a Source Data file.

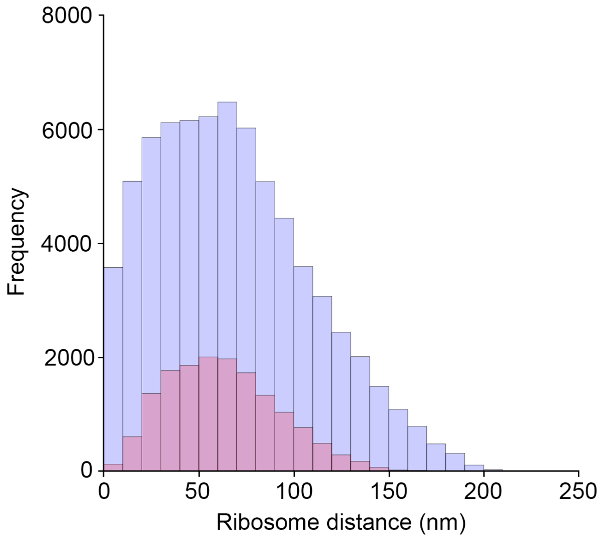

Supplementary Figure 9 – Distribution of ribosome distances to the milling edge before and after 3D classification. The frequency of putative-ribosomes identified with crYOLO (blue) and 80S ribosomes after 3D classification (red) based on the ribosome distance in nanometers (bin size: 10 nm). Source data are provided as a Source Data file.

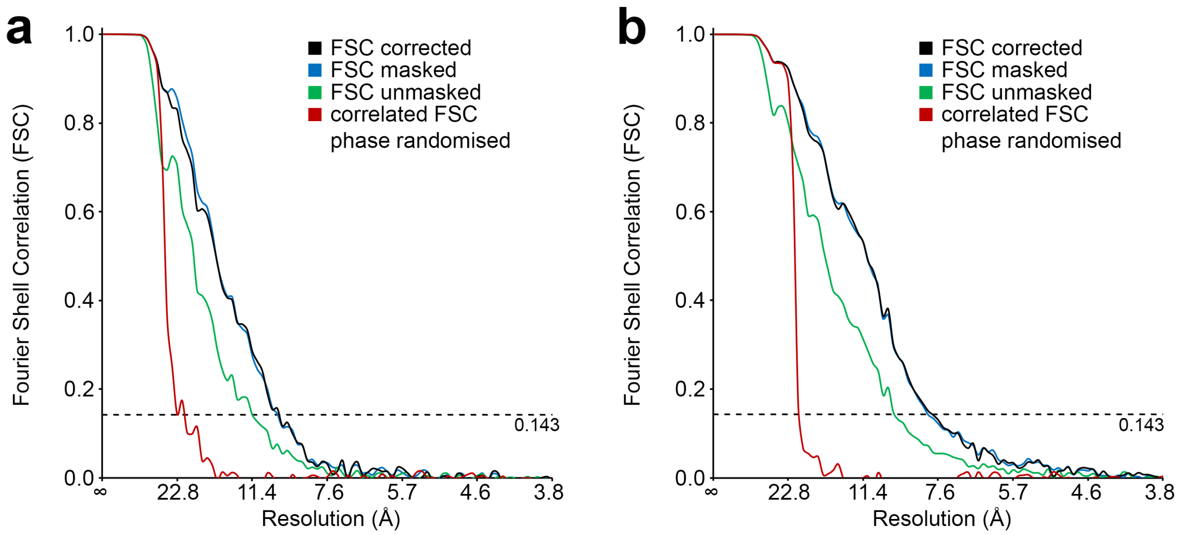

Supplementary Figure 10 – FSC curves for ribosome structures from within 30 nm distance from lamella edge and in the centre. FSC curves are shown for ribosomes structures determined from: (a) (b) 1748 between 15 and 30 nm from the milling surfaces and 1748 ribosomes more than 30 nm of the surfaces, matched to be from the same tomograms. Resolutions are 9.8 Å and 8.0 Å, respectively.

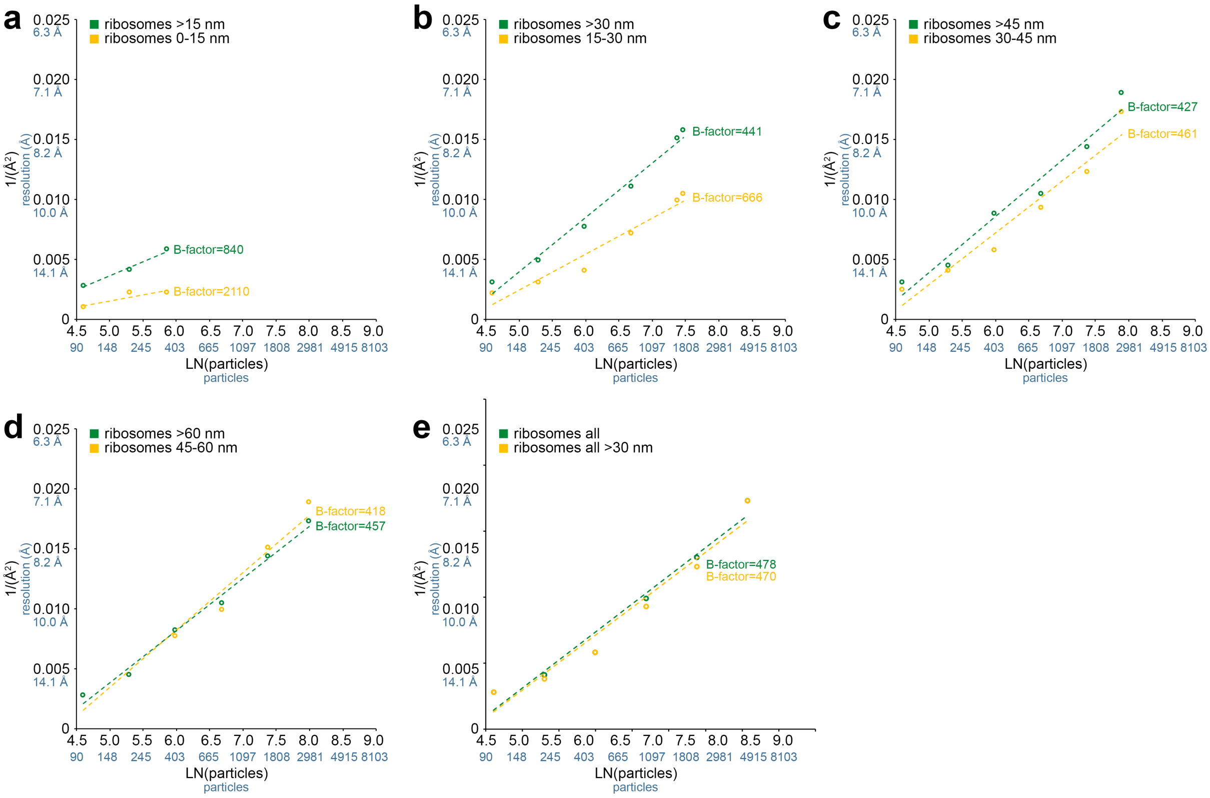

Supplementary Figure 11 – Ribosome B-factors as a function of distance from the PFIB milling surfaces. B-factors plots for ribosomes at different distances from the PFIB milling surfaces, with controls using the same number of particles further away from the surfaces, matched to be from the same tomograms where possible. Source data are provided as a Source Data file.

Supplementary Videos

Supplementary Video 1. Tomographic volume of the slice shown in Fig. 1h. Scalebar: 100 nm.

Supplementary Video 2. Video of the 3-dimensional volume local resolution map shown in Fig. 3b. Scale bar is 10 nm

Supplementary Video 3. Sub volume average density map of the ribosomal L7subunit obtained in this study overlaid with the previously obtained structure of isolated human ribosomes (PDB: 4UG0[53]). Scale bar is 1 nm.
