# Supplementary material for: Plasma FIB milling for the determination of structures in situ
Source: Nat Commun. 2023 Feb 6;14:629. doi: 10.1038/s41467-023-36372-9 (PMC9902539; doi:10.1038/s41467-023-36372-9)
Supplement: Supplementary file 3 — Description of additional Supplementary File [file 41467_2023_36372_MOESM3_ESM.pdf]

## Descriptions of additional supplementary files

**Supplementary Video 1.** Tomographic volume of the slice shown in Fig. 1h. Scalebar: 100 nm.

**Supplementary Video 2.** Video of the 3-dimensional volume local resolution map shown in Fig. 3b. Scale bar is 10 nm

**Supplementary Video 3.** Sub volume average density map of the ribosomal L7subunit obtained in this study overlaid with the previously obtained structure of isolated human ribosomes (PDB: 4UG0[53]). Scale bar is 1 nm.

**Supplementary Software**. RiboDist is a programme developed to enable the calculation of the distance between particles (e.g. ribosomes) and the edges of a volume, such as those found at the edges of a focused ion beam-generated lamella. It enable the particles to be parsed into separate files that can be used for subsequent sub-tomogram averaging.
